# The Clinical Characteristics and Predictors of Refractory *Mycoplasma pneumoniae* Pneumonia in Children

**DOI:** 10.1371/journal.pone.0156465

**Published:** 2016-05-26

**Authors:** Yuanyuan Zhang, Yunlian Zhou, Shuxian Li, Dehua Yang, Xiling Wu, Zhimin Chen

**Affiliations:** Department of Pulmonology, Children’s Hospital, Zhejiang University School of Medicine, Hangzhou, P.R. China; University of Verona, ITALY

## Abstract

**Objective:**

To analyze the clinical characteristics of refracory *Mycoplasma pneumoniae* pneumonia (RMPP), and explore the related factors predicting RMPP.

**Methods:**

Retrospective analysis was performed on 634 children with *Mycoplasma pneumoniae* pneumonia (MPP) hospitalized in our hospital between January 1, 2011 and December 31, 2014. The clinical features, laboratory data, radiological findings between the RMPP group and the general *Mycoplasma pneumoniae* pneumonia (GMPP) group were compared and the predictive values of related factors were analyzed.

**Results:**

The median age of the RMPP patients (n = 145) was much older than that of the GMPP patients (n = 489) (P<0.01). We also found more severe presentations, higher incidence of extra-pulmonary complications and more serious radiological findings in RMPP group, which needed oxygen more often, longer antibiotics administration and intensive care (P<0.05). Meanwhile, the levels of C-reactive protein (CRP), lactic dehydrogenase (LDH), immunoglobulin A (IgM), interleukin (IL)-6, IL-10, interferon gamma (IFN-γ) and the percentage of neutrophils, CD8+ in RMPP group were significantly higher than those in GMPP group (P<0.05); while the levels of prealbumin (PAB) were lower than that in GMPP group (P<0.01). In ROC curve analysis, the percentage of neutrophil, CRP, LDH, PAB, IL-6, IL-10 and IFN-γ were useful for differentiating patients with RMPP from those with GMPP. Multiple logistic regression analysis showed that the CRP≥16.5mg/L, LDH ≥417IU/L and IL-6 ≥14.75pg/ml were significant predictors regarding to RMPP.

**Conclusions:**

CRP≥16.5mg/L, LDH ≥417IU/L and IL-6 ≥14.75pg/ml might be the significant predictors of RMPP in children, which can aid in early recognition of RMPP.

## Introduction

*Mycoplasma pneumoniae* (MP) is one of the most prevalent pathogens causing community-acquired pneumonia (CAP) in children [1, 2]. Prior studies showed that MP might account for as many as 40% of CAP cases and 18% of these patients require hospitalization [3].

Although *Mycoplasma pneumoniae* pneumonia (MPP) is usually considered as a self-limited disease, sometimes it may cause various pulmonary and extra-pulmonary complications such as bronchiolitis obliterans, necrotizing pneumonia, encephalitis, arthritis, pericarditis, hemolytic anemia, and develop into a severe life-threatening pneumonia [4–11]. For children, macrolides are the first-choice antibiotics for MP infections. However, there still are some cases showing clinical and radiological deterioration despite of macrolide antibiotic therapy for 7 days or longer [12, 13] to be defined as refractory *Mycoplasma pneumoniae* pneumonia (RMPP). Therefore, it is important for clinicians to recognize RMPP earlier and grasp the appropriate opportunity for reasonable therapy.

In order to explore the predictive values of the independent related factors of RMPP, we retrospectively analyzed the cases of MPP hospitalized in our hospital between January 1, 2011 and December 31, 2014, then compared the differences of clinical features, laboratory data and radiological findings between RMPP and general *Mycoplasma pneumoniae* pneumonia (GMPP) children.

## Methods

### Study population

In this study we retrospectively collected the data of patients with MMP who admitted to Children’s hospital, Zhejiang University School of Medicine between January 1, 2011 and December 31, 2014. All the patients had signs and symptoms indicative of pneumonia on admission, including fever, cough, abnormal lung auscultation and a new infiltrate on chest radiograph [14]. The diagnosis of MP infection was based on the positive results for serologic test (MP IgM positive and antibody titer≥1:160) while having the positive results for MP polymerase chain reaction (PCR) tests of nasopharyngeal secretions. The diagnosis of RMPP was based on the presence of persistent fever and clinical as well as radiological deterioration after azithromycin treatment for 7 days or longer [12, 13]. All patients were excluded with other respiratory tract infections and tuberculosis by following tests: protein purified derivative (PPD), blood cultures, pleural effusion cultures, nasopharyngeal aspirate/swab cultures, nasopharyngeal aspirate/swab for virus antigens detection (respiratory syncytial viruses, influenza viruses, metapneumovirus, adenovirus, and parainfluenza virus), and serology for Chlamydia pneumoniae (CT) and Legionella pneumophila (LG). Patients who received corticosteroids before admission or had underlying diseases such as asthma, recurrent respiratory tract infection, chronic cardiac and pulmonary disease, rheumatic diseases and immunodeficiency were also excluded.

### Data collection

Demographic, clinical information, laboratory data and radiological findings were retrospectively collected from all children who were included in the study. Nasopharyngeal aspirate/swab specimens were routinely collected within 24 hours of admission. Respiratory specimens were tested for bacterial culture, virus using direct immunofluorescence assays and MP using PCR. Peripheral blood samples were obtained on admission for the determination of the complete blood count, C-reactive protein (CRP), lactate dehydrogenase (LDH), prealbumin (PAB), immunoglobulins, subpopulations of T lymphocytes, specific antibody to MP, CT, LG, and cytokines including interleukin (IL)-2, IL-4, IL-6, IL-10, tumor necrosis factor alpha (TNF-α) and interferon gamma (IFN-γ). Blood culture was also performed on admission. The blood count and CRP were detected every 2–3 days thereafter if abnormal. The majority of patients were treated with macrolide (azithromycin or erythromycin) for 7–14 days, partly with a broad-spectrum antibiotic (ceftriaxone or amoxacilin) simultaneously. All patients underwent chest radiography before admission or during hospitalization, presenting unequivocal focal or segmental infiltration with or without pleural effusion. Large lesion was defined when the extent of infiltration on chest radiography was more than 1/3 of the lung [15]. All children enrolled were followed until discharging. Body temperature and respiratory tract signs and symptoms of subjects were examined at study entry and every 8 hour thereafter. A febrile day was defined as a day during which the body temperature exceeded 38.0°C at least once [16]. During the hospitalization, we also evaluated the extra-pulmonary complications (liver function abnormalities, myocarditis, encephalitis, rash, proteinuria, hemolytic anemia and arthritis) of patients [17]. Hypoxia was defined as any recorded oxygen saturation of <92% by pulse oximetry, measured on room air [18]. The indication for oxygen therapy and mechanical ventilation were evaluated according to the Guidelines for management of community acquired pneumonia in children in China [12].

### Ethics

The study was approved by the ethics committee of the Children’s Hospital, Zhejiang University School of Medicine. And the data from patients were analyzed anonymously.

### Statistical Analysis

Statistical analyses were performed using SPSS software (version 15.0). Normal distribution data were expressed as mean ± SD ($\bar{x}$±s). Independent-Samples T-test was used to compare these data. Skewed distribution data were expressed as median values (25th-75th interquartile ranges). The comparisons were made by the Mann-Whitney U-test. And Chi-squared tests were used to compare categorical data. Receiver operating characteristic (ROC) curves were operated to evaluate candidate indicators with regards to the refractory assessment of patients with MMP. Logistic regression analysis was performed to select the variables associated with the RMPP. Statistical significance was defined as P<0.05.

## Results

### General information of patients

A total of 634 patients who were diagnosed with MPP in our hospital from January 1, 2011 to December 31, 2014 were enrolled in the study. All patients had positive results of PCR test and serological detection, and had not fulfilled exclusive criteria. The median age was 3.9 (2.1~6.8) years with a female-to-male ratio of 0.81. All patients were previously healthy, without underlying disease. According to the diagnostic criteria of RMPP [12, 13], patients were divided into two groups, GMPP and RMPP. 489 patients were in the GMPP group (280 males, 209 females), with the median age of 3.4 (1.9~6.3) years. 145 patients were in the RMPP group (70 males, 75 females), with the median age of 5.9 (3.8~8.0) years. The median age of the RMPP patients was much older than that of the GMPP patients (P<0.01), but no difference in gender distribution was found between the two groups.

### Clinical and laboratory characteristics of GMPP and RMPP patients

567 (89.4%) patients presented fever in our study, but fever was more common in RMPP group than that in the GMPP group (P<0.01). We also compared other symptoms and respiratory signs between the two groups. The incidences of tachypnea, chest tightness, chill and decreased unilateral lung sound were significantly higher in the RMPP patients than those in the GMPP patients (P<0.01, P<0.05, P<0.05, P<0.01, respectively). The incidence of wheezing was significantly lower in the RMPP group than that in the GMPP group (P<0.01). Of the 634 patients, extra-pulmonary complications were found in 121 cases (19.1%), including digestive system in 28, cardiovascular system in 29, nervous system in 2, urinary system in 6, joint system in 1, rash in 50, and hematological system in 5. The incidence of extra-pulmonary complications was 40.0% in RMPP group and 12.9% in GMPP group, with a significant difference (P<0.01) (Table 1).

**Table 1:** Clinical characteristic of GMPP and RMPP patients.

Clinical information	GMPP (n = 489)	RMPP (n = 145)	*P*-value
Age, years	3.4 (1.9~6.3)	5.9 (3.8~8.0)	0.000
Sex (male/female)	280/209	70/75	0.058
Clinical presentation, n (%)			
Fever	422 (86.3%)	145 (100%)	0.000
Cough	489 (100%)	145 (100%)	1.000
Tachypnea	31 (6.3%)	35 (24.1%)	0.000
Chest tightness	2 (0.4%)	4 (2.8%)	0.027
Wheezing	70 (14.3%)	2 (1.4%)	0.000
Chill	9 (1.8%)	9 (6.2%)	0.010
Extra-pulmonary complications	63 (12.9%)	58 (40.0%)	0.000
Physical examination, n (%)			
Rales	332 (67.9%)	86 (59.3%)	0.058
Decreased unilateral lung sound	59 (12.1%)	60 (41.4%)	0.000
Length of fever, days	7.0 (3.3~10.0)	13.0 (11.0~16.0)	0.000
Length of stay, days	6.0 (4.0~7.0)	9.0 (7.0~11.5)	0.000
Management			
Length of antibiotic therapy days	11 (8~11)	16 (14~18)	0.000
Oxygenotherapy, n (%)	26 (5.3%)	40 (27.6%)	0.000
Intensive care unit, n (%)	0 (0%)	4 (2.8%)	0.003

Data are presented as number (percentage), median (25^th^-75^th^ percentile).

Laboratory data and radiological findings in RMPP and GMPP patients were summarized in Tables 2 and 3. Regarding the laboratory examinations, the median levels of CRP, LDH, and the median percentage of peripheral neutrophils in children with RMPP were significant higher than those in children with GMPP (P<0.01); Patients with RMPP had significant lower level of PAB, compared to that with GMPP (P<0.01). Meanwhile, we found significant higher levels of cytokines (including IL-6, IL-10, IFN-γ), IgA and higher percentage of CD8^+^ in the RMPP group when compared with the GMPP group (P<0.01, P<0.01, P<0.05, respectively). However, the median values of WBC, IgM, IgG, CD3^+^, CD4^+^, IL-2, IL-4 and TNF-α did not differ significantly between the two groups.

**Table 2:** Laboratory characteristic of GMPP and RMPP patients.

Laboratory information	GMPP (n = 489)	RMPP (n = 145)	*P*-value
White blood cell (×10^9^/L)	8.22 (6.27~10.56)	7.60 (5.71~9.90)	0.106
Neutrophil, %	56.7 (43.4~65.6)	73.1 (65.3~78.5)	0.000
C-reactive protein (CRP), mg/L)	6 (1~14)	36 (13~90)	0.000
Lactatedehydrogenase (LDH), IU/L	366 (310~459)	537 (419~666)	0.000
Prealbumin (PAB), g/L	0.12 (0.10~0.16)	0.08 (0.06~0.11)	0.000
Total Immunoglobulin (Ig), g/L			
IgG	9.36 (7.24~11.20)	8.96 (7.56~11.32)	0.823
IgA	0.85 (0.51~1.31)	1.20 (0.77~1.54)	0.000
IgM	1.54 (1.11~2.18)	1.66 (1.16~2.43)	0.138
Subpopulations of T lymphocytes, %			
CD3^+^	61.48 (54.52~69.69)	61.97 (55.68~71.64)	0.243
CD4^+^	33.97 (28.26~39.29)	34.26 (27.12~39.99)	0.909
CD8^+^	21.16 (16.43~25.34)	22.63 (17.40~27.99)	0.040
Cytokines, pg/ml			
Interleukin 2 (IL-2)	2.6 (1.7~3.7)	2.5 (1.8~3.5)	0.883
IL-4	2.8 (2.2~3.4)	2.9 (2.2~3.5)	0.929
IL-6	9.7 (4.7~24.9)	34.2 (14.3 ~87.3)	0.000
IL-10	4.2 (3.1~6.1)	6.7 (4.7~10.0)	0.000
Tumor necrosis factor alpha (TNF-α)	2.9 (2.0~4.1)	3.0 (1.9~4.7)	0.866
Interferon gamma (IFN-γ)	7.9 (4.6 ~12.2)	16.3 (8.3~49.3)	0.000

Data are presented as the median (25^th^-75^th^ percentile).

**Table 3:** Radiological features of GMPP and RMPP patients.

Radiological features	GMPP (n = 489)	RMPP (n = 145)	*P*-value
% Patients with large lesions	168 (34.4%)	105 (72.4%)	0.000
% Patients with pulmonary complications			
Pleural effusion	54 (11.0%)	70 (48.3%)	0.000
Lobar atelectasis	54 (11.0%)	38 (26.2%)	0.000
Pulmonary consolidation	19 (3.9%)	32 (22.1%)	0.000
Pleural thickening	17 (3.5%)	17 (11.7%)	0.000
Necrotizing pneumonia	2 (0.4%)	2 (1.4%)	0.226

Data are presented as number (percentage).

In addition to laboratory data, radiological findings were more severe in the RMPP group than that in the GMPP group (Table 3). 72.4% of the patients in the RMPP group showed large lesions versus 34.4% in the GMPP group (P<0.01). And there were significant differences between the two groups in the incidence of pulmonary complications, including pleural effusion (48.3% versus 11.0%, P<0.01), lobar atelectasis (26.2% versus 11.0%, P<0.01), consolidation (22.1% versus 3.9%, P<0.01) and pleural thickening (11.7% versus 3.5%, P<0.01). But the difference in the incidence of necrotizing pneumonia did not reach statistical significance (P>0.05).

Concerning the clinical course, we found that the median length of stay days was 9.0 (7.0~11.5) days in the RMPP group and 6.0 (4.0~7.0) days in the GMPP group (P<0.01), and the median length of fever was 13.0 (11.0~16.0) days in the RMPP group versus 7.0 (3.3~10.0) days in the GMPP group (P<0.01) (Table 1). All patients were treated with antibiotics. The median length of antibiotic therapy was 16 (14~18) days in RMPP group and 11 (8~13) days in the GMPP group, with a significant difference (P<0.01). Additionally, the proportion of patients required oxygentherapy and intensive care unit in the RMPP group was higher than that in the GMPP group (P<0.01).

### Predictive values of the independent correlation factors in patients with RMPP

To explore the predictive values of laboratory date for RMPP, receiver operator characteristic (ROC) curves were made and the cut-off values with maximum sensitivities and specificities were determined. Analysis of these ROC curves showed that the percentage of neutrophil, CRP, LDH, PAB, IL-6, IL-10 and IFN-γ were useful for differentiating patients with RMPP from those with GMPP (Table 4). When the cut-off values for the percentage of neutrophil, CRP, LDH, PAB, IL-6, IL-10 and IFN-γ were set at 68.6%, 16.5mg/L, 417IU/L, 0.095g/L, 14.75pg/ml, 4.65 pg/ml and 15.50 pg/ml, respectively, the sensitivity and specificity in differentiating RMPP from GMPP were 68.4% and 81.1%, 74.7% and 77.2%, 79.7% and 65.0%, 75.7% and 63.7%, 83.5% and 63.4%, 75.9% and 61.0%, 55.7% and 84.6%, respectively.

**Table 4:** Predictive values of the independent correlation factors in patients with RMPP.

Independent factors	Cutoff value	Sensitivity	Specificity	AUC	P-value
Age, years	3.875	0.745	0.564	0.674	0.000
Neutrophil, %	68.6	0.684	0.811	0.803	0.000
C-reactive protein (CRP), mg/L	16.5	0.747	0.772	0.817	0.000
Lactatedehydrogenase (LDH), IU/L	417	0.797	0.650	0.772	0.000
Prealbumin (PAB), g/L	0.095	0.757	0.637	0.747	0.000
Immunoglobulin A (IgA), g/L	0.66	0.861	0.402	0.636	0.000
CD_8_^+^, %	25.05	0.430	0.768	0.603	0.006
Interleukin 6 (IL-6), pg/ml	14.75	0.835	0.634	0.758	0.000
IL-10, pg/ml	4.65	0.759	0.610	0.722	0.000
Interferon gamma (IFN-γ), pg/ml	15.50	0.557	0.846	0.724	0.000

AUC: area under the ROC curve; Cut-off value: the value on the ROC curve is closest to the upper right to take maximum sensitivity and specificity; P-value: the AUC value of the independent factors compared to ROC curve reference value 0.5.

### Multiple logistic regression analysis for the related factors predicting the RMPP

Multiple logistic regression analysis of 634 cases was performed to assess predictors which allowed the differential diagnosis of RMPP and GMPP. The CRP≥16.5mg/L, LDH ≥417IU/L and IL-6 ≥14.75pg/ml were significantly predictive regarding the differentiation between the two groups, with the odd ratio (OR) values of 2.023, 2.185, and 2.314, respectively in Table 5.

**Table 5:** Stepwise logistic regression analysis for the related factors predicting the RMPP.

Variable	B	S.E.	Wald	P-value	OR	95%CI	
						Lower	Upper
C-reactive protein (CRP) ≥16.5mg/L	0.705	0.330	4.570	0.033	2.023	1.060	3.861
Lactatedehydrogenase (LDH) ≥417IU/L	0.782	0.319	5.998	0.014	2.185	1.169	4.084
Interleukin 6 (IL-6) ≥14.75pg/ml	0.839	0.333	6.341	0.012	2.314	1.204	4.446

## Discussion

MP is one of the major pathogens causing CAP in children, especially in Asian. Although MP infection was traditionally thought to be a self-limited process, more and more severe cases even fatal cases of MP infections were reported in recent years [4–11]. Cases of RMPP, which display clinical and radiological progression after macrolide therapy for 7 days or longer, are reported increasingly [13, 19, 20]. So it is essential for pediatricians to recognize RMPP early, treat it promptly and prevent the progress of the disease. To our knowledge, there is still a paucity of data about clinical characteristics and risk factor of RMPP in a large number of cases until now.

In the present, retrospective, observational study, 634 patients with MPP were enrolled, and the different clinical characteristics between the RMPP patients and GMPP patients were compared. According to the diagnostic criteria of RMPP [12, 13], 145 cases were diagnosed as RMPP, while 489 were GMPP. Firstly, we found that the median age of the RMPP patients was much older than that of the GMPP patients (5.9 (3.8~8.0) versus 3.4 (1.9~6.3), P<0.01), which was in accordance with the previous reports [20, 21]. The immune system in older children was relatively more mature than that in younger children, and inappropriate immune response to MP was easy to produce excessive inflammatory reaction, which might contribute to the development of RMPP.

Secondly, more severe signs and symptoms and higher incidence of extra-pulmonary complications were found in the RMPP group than those in the GMPP group (P<0.05). We also found that patients with RMPP showed longer median length of stay days, longer median length of fever days and longer median length of antibiotic therapy days than those of patients with GMPP (P<0.01). Moreover, the proportion of patients required oxygen therapy and intensive care unit in the RMPP group was higher than that in the GMPP group (P<0.01). These results implied that RMPP was refractory to treatment and could result in prolonged clinical course.

Additionally, our study showed that 72.4% of the patients in the RMPP group had large lesions of radiological manifestation versus 34.4% in the GMPP group (P<0.01). And higher incidence of pleural effusion, lobar atelectasis, consolidation and pleural thickening were found in the RMPP group than in the GMPP group (P<0.01). The diversification of imaging findings might due to direct microbe effect and host strong immune response and these significant radiographical evidences of lung damage were in compliance with the complicated course.

Although the underlying mechanisms are still uncertain, the macrolide-resistant MP infection and excessive immunological inflammation are most commonly proposed [22–26]. Cao et al [27] reported that the resistance rate of MP in adult patients with respiratory tract infection was reached to 69% in 2010. But in our previous study we found higher prevalence (87.7%) of macrolide-resistance in MPP patients, and there were no significant difference of resistance rate of MP between the GMPP group and the RMPP group [17]. So we think that cell-mediated immunological response plays an important role in the progression of MPP. Tanaka et al. demonstrated that the levels of serum interleukin-18, which promotes Th1 cytokine responses, in patients with severe MP were higher than those in mild cases [21]. Here, we compared some other laboratory markers between the RMPP group and GMPP group. Several serum characteristics including markedly increased CRP levels, LDH levels and percentage of neutrophils were observed to be associated with RMPP, which was well consistent with other reports [20, 28–30]. Additionally, our study firstly demonstrated a significant lower level of PAB in the RMPP patients, which perhaps also indicated severe systemic inflammatory response to MP infection.

Inflammatory cytokines were also involved in the immunopathogenesis of MP infection [31–33]. In our study, we found that higher levels of IL-6, IL-10, IFN-γ in the RMPP group than those in the GMPP group (P<0.01). Meanwhile, higher levels of IgA and higher percentage of CD8^+^ in the RMPP patients were detected when compared with the GMPP group (P<0.01, P<0.05, respectively). The excessive inflammation reaction may lead to release of cytokines and immune disorder, which is might related to the severity of RMPP in children.

To investigate potential factors that may allow differentiation between RMPP and GMPP, we analyzed age and some laboratory markers, which were significantly different between the two groups using ROC curve. In our study, we found that the area under the curve for seven independent factors, including the percentage of neutrophil, CRP, LDH, PAB, IL-6, IL-10 and IFN-γ were above 0.7 in ROC curve analysis, indicating fair discriminative power for predicting RMPP. The optimal cutoff value for these seven factors was 68.6%, 16.5mg/L, 417IU/L, 0.095g/L, 14.75pg/ml, 4.65 pg/ml and 15.50 pg/ml, respectively. Furthermore, basing on the cutoff values of these seven factors, multiple logistic regression analysis were made to improve the predicted accuracy. We found that the LDH ≥417IU/L, CRP≥16.5mg/L and IL-6 ≥14.75pg/ml were significant predictors of RMPP. LDH was associated with many pulmonary diseases, such as obstructive diseases, microbial pulmonary diseases, and interstitial lung diseases [34, 35]. Several studies [21, 29, 30] also found that serum LDH was elevated in RMPP. In our study, we found that the area under the curve for LDH was 0.772 in ROC curve analysis, indicating fair discriminative power for predicting RMPP. The optimal cutoff for LDH was 417 IU/L, with a sensitivity of 79.7% and specificity of 65.0%, which was similar with previous repot [29]. And stepwise logistic regression analyzed that the serum LDH (odds ratio of 2.185, 95% CI 1.169~4.084, P = 0.014) was significant meaningful. CRP is a gross biochemical index of inflammation and is used commonly in the clinical setting. CRP reflected the acute severe systemic inflammatory reactions to MP infection, and was suggestive of a well-developed immune system. Liu et al showed that the cutoff value of CRP for RMPP was 40mg/L [30]. The cutoffs were greater than in our study. The main reason may be that their results were obtained from a small case series, and they had more serious illnesses. In this study, the optimal cutoff point for CRP was 16.5mg/L, with a sensitivity of 74.7% and specificity of 77.2%, and the odds ratio of logistic regression analysis was 2.023. These indicate theirs clinical utility in identifying patients at high risk for RMPP. More interestingly, this is the first report demonstrated that IL-6 ≥14.75pg/ml might help us to differentiate RMPP from GMPP. However, the measurement of IL-6 is relatively costly and has a relatively high inter-laboratory variation in accuracy of the measure. So IL-6 is not a parameter commonly measured in the clinical setting but limited to research purposes.

This study has several limitations. Firstly, it was a retrospective study, and therefore there may have been some selection bias. Secondly, there may be some cases in which the patients had a combined MP and other pathogens infection which cannot be detected, which might result in RMPP. Thirdly, the distribution of patients between the two groups is not matching, which might affect the statistic results. Fourthly, in spite of the potential usefulness of using a biochemical parameter for categorizing RMPP from MPP patients, the sensitivity and the specificity of the variables identified are relatively modest. Therefore a large number of patients with RMPP are needed to be enrolled and a further prospective study is needed to be carried out.

In conclusion, older children are more prone to developing RMPP, leading to more severe presentations, higher incidence of extra-pulmonary complications and more serious radiological findings. Clinician might use the LDH, CRP and/or IL-6 cut off (CRP≥16.5mg/L, LDH ≥417IU/L and IL-6 ≥14.75pg/ml) for identifying children at higher risk of RMPP in comparison to all the other clinical, radiological and biochemical information.

## Supporting Information

S1 File STROBE Statement—Checklist of items that should be included in reports of case-control studies. (DOC)
